# MyD88 and TRIF mediate the cyclic adenosine monophosphate (cAMP) induced corticotropin releasing hormone (CRH) expression in JEG3 choriocarcinoma cell line

**DOI:** 10.1186/1477-7827-7-74

**Published:** 2009-07-17

**Authors:** Andy Uh, Charles F Simmons, Catherine Bresee, Nasif Khoury, Adrian F Gombart, Richard C Nicholson, Hande Kocak, Ozlem Equils

**Affiliations:** 1 Ahmanson Department of Pediatrics, Room 4221, Steven Spielberg Pediatric Research Center, Burns and Allen Research Institute, Cedars-Sinai Medical Center, David Geffen School of Medicine at UCLA, Cedars-Sinai Medical Center, Los Angeles, CA 90048, USA; 2 Samuel Oschin Comprehensive Cancer Institute Biostatistics Core, Cedars-Sinai Medical Center, Cedars-Sinai Medical Center, Los Angeles, CA 90048, USA; 3 Linus Pauling Institute; Department of Biochemistry and Biophysics; ALS 2011, Oregon State University; Corvallis, OR 97331-7305, USA; 4 Mothers and Babies Research Center, Hunter Medical Research Institute, John Hunter Hospital, Newcastle, Australia; 5 Department of Human Genetics, University of Michigan, Ann Arbor, MI, USA

## Abstract

**Background:**

Classically protein kinase A (PKA) and transcription factor activator protein 1 (AP-1) mediate the cyclic AMP (cAMP) induced-corticotrophin releasing hormone (CRH) expression in the placenta. However enteric Gram (-) bacterial cell wall component lipopolysaccharide (LPS) may also induce-CRH expression via Toll like receptor (TLR)4 and its adaptor molecule Myd88. Here we investigated the role of MyD88, TRIF and IRAK2 on cAMP-induced CRH promoter activation in JEG3 cells in the absence of LPS/TLR4 stimulation.

**Methods:**

JEG3 cells were transfected with CRH-luciferase and Beta-galactosidase expression vectors and either empty or dominant-negative (DN)-MyD88, DN-TRIF or DN-IRAK2 vectors using Fugene6 (Roche). cAMP-induced CRH promoter activation was examined by using a luminometer and luciferase assay. Calorimetric Beta-galactosidase assays were performed to correct for transfection efficiency. Luciferase expression vectors of cAMP-downstream molecules, CRE and AP-1, were used to further examine the signaling cascades.

**Results:**

cAMP stimulation induced AP-1 and CRE promoter expression and led to dose-dependent CRH promoter activation in JEG3 cells. Inhibition of MyD88 signaling blocked cAMP-induced CRE and CRH promoter activation. Inhibition of TRIF signaling blocked cAMP-induced CRH but not CRE expression, while inhibition of IRAK2 did not have an effect on cAMP-induced CRH expression.

**Conclusion:**

MyD88 and TRIF exert direct regulatory effect on cAMP-induced CRH promoter activation in JEG3 cells in the absence of infection. MyD88 most likely interacts with molecules upstream of IRAK2 to regulate cAMP-induced CRH expression.

## Background

Cyclic AMP-protein is a second messenger that mediates the physiologic responses to a number of hormones, neurotransmitters, and drugs. β-adrenoceptor agonists, prostaglandin E_2 _(PGE_2_) activate adenyl cyclase; which catalyzes the conversion of ATP to cAMP. cAMP then binds and activates protein kinase A (PKA), which phosphorylates substrates that regulate key cellular functions such as ion channels, contractile proteins and transcription factors.

One of the molecules induced by cAMP is corticotropin releasing hormone (CRH). In the placenta cAMP induces CRH expression via PKA, cAMP response element (CRE) and transcription factor activating protein (AP)-1 signaling [1]. CRH is then thought to lead to positive feedback mechanisms with fetal cortisol and dehydroepiandrosterone (DHEA) production, fetal maturation and initiation of the parturition.

Infection is a well known risk factor associated with preterm delivery [2], and innate immune system receptors, Toll like receptors, via adaptor molecule myeloid differentiation primary response (MyD)88 [3] and TIR-domain-containing adapter-inducing interferon-beta (TRIF) [3] and their down stream signaling molecule interleukin-1 receptor-associated kinase (IRAK)2 are expressed in uterus and placenta, and mediate the infection associated inflammatory responses [4-6]. We have previously shown that MyD88 mediates the Toll like receptor (TLR)4-lipopolysaccharide (LPS)-induced CRH expression in JEG3 choriocarcinoma cell line [7]. In those experiments we used cAMP as the positive control and observed that inhibition of MyD88 signaling, in the absence of LPS stimulation, blocked the cAMP-induced CRH promoter activation as well. Here, our aim is to further examine the role of MyD88 and TRIF in cAMP-induced CRH promoter activation in the absence of infection or TLR stimulation.

## Methods

### Cell lines and reagents

JEG3 choriocarcinoma cell line was obtained from American Type Tissue Culture Collection (Manaaas, VA) and cultured in MEM (Invitrogen Life Technologies, Carlsbad, CA) supplemented with 10% fetal bovine serum, 10 mM HEPES, 1 mM sodium pyruvate and 100 nM of penicillin/streptomycin (Invitrogen Life Technologies). A cell permeable cyclic AMP (cAMP) analog, 8-bromoadenosine 3',5' cAMP was obtained from Sigma-Aldrich (St. Louis, MO).

### Expression vectors

The CRH-luciferase vector, pGL3-CRH 663, was characterized and described previously [8]. Dominant negative (DN) cDNA constructs of MyD88 and interleukin-1 receptor-associated kinase (IRAK)2 have been characterized and described previously [9]. The NH_2_-terminally deleted DN-MyD88 coding for amino acids 152–296 inhibits IL1 induced NF-kB activation [9]. IRAK2 is downstream to MyD88 and DN-IRAK2 coding for aa 1–96 inhibits MyD88 induces signaling [9]. The pcDNA3 empty vector and pCMV-β-galactosidase vectors have been described previously [10]. Luciferase data obtained from the cells transfected with pcDNA3 empty vector was used to assess the specificity of dominant negative vectors (DN-MyD88, DN-TRIF) to suppress cAMP-induced luciferase activity. The viral cAMP response element (CRE)-luciferase vector was described by Giebler et al [11]. This vector contains CRE and GC-rich flanks form a critical DNA element (called the viral CRE) that is obligatory for human T cell leukemia virus type 1 (HTLV-1) protein Tax transactivation [11]. Dr. Shizuo Akira generated the dominant negative TIR domain-containing adapter inducing IFN-beta (TRIF) by truncating the full length 712 aa TRIF to 162 aa Toll/Interleukin-1 receptor domain (TIR)-only domain [12]. The AP-1 luciferase construct was kindly obtained from Dr. Phillip Koeffler (Cedars-Sinai Medical Center, UCLA School of Medicine).

### Transfection of JEG3 cells

JEG3 cells were plated at a concentration of 50,000 cells/well in 24-well plates and cultured in MCDB-131 with 10% fetal bovine serum overnight. Cells were co-transfected the following day with FuGene6 Transfection Reagent following the manufacturer's instructions. The Roche Fugene6 transfection system is routinely used by our laboratory and others, and does not affect the cell viability or induce cytokine production in the transfected cells [10]. The reporter genes CRH-Luciferase (0.5 μg) and either empty vector or dominant negative mutants of MyD88 and TRIF were transfected into the JEG3 cells. Reporter gene CRE-luciferase (0.5 μg) was transfected to assess the effect of TLR stimulation on CRE expression. pCMV-β-galactosidase cDNA (0.1 μg) was transfected to normalize the results for transfection efficiency as described earlier [10].

After overnight transfection, the cells were stimulated with various concentrations of cAMP. CRH-luciferase cDNA transfected JEG3 cells were stimulated with cAMP for 5 hrs or 20 hrs in separate sets of experiments. Cells were then lysed and luciferase activity was measured with a Promega kit (Promega, Madison, WI) and a luminometer. The adequacy of the transfection was assessed by co- transfecting the cells with β-galactosidase vector and performing calorimetric galactosidase assay. This is a very well established and accepted method of assessing the transfection efficiency. The cells transfected with β-galactosidase are also considered to be transfected with the vector in question, in our case CRH-luciferase vector. β-galactosidase activity was determined by calorimetric method as described earlier [10]. The luciferase data was corrected for transfection efficiency by dividing the luciferase measurement with the galactosidase measurement. This result was expressed as the relative light unit (RLU).

### Statistical analysis

Each experiment was performed in triplicate or quadruplicate and repeated at least three independent times. To study the effect of the varying combinations and concentrations of cAMP and the DNA-vectors across each assay, one-way ANOVA was used to examine the difference in luciferase units across the different treatment groups. Tukey's test was then used to compare the average luciferase units between each of the treatment groups against the control while properly adjusting for multiple comparisons. The data presented are the results of a representative experiment.

## Results

### cAMP induces CRH promoter activation in the JEG3 cells

**Figure 1:**
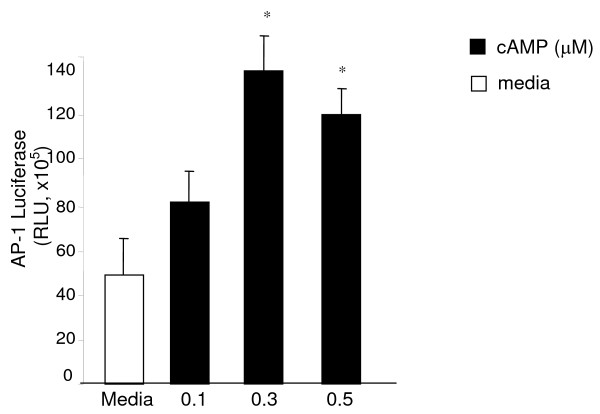
**The JEG3 cells were transiently transfected overnight with AP-1 luciferase and β-galactosidase expression vectors**. The cells were stimulated with cAMP for 5 hr and AP-1 promoter activation was assessed by performing luciferase assay. Calorimetric β-galactosidase assay was performed to correct for the transfection efficiency. Luciferase activity was expressed as relative light unit (RLU). (*p < 0.01 compared to media treated cells). Each experiment was performed in triplicate or quadruplicate and repeated at least three independent times.

cAMP is known to induce CRH expression in the trophoblasts. We first confirmed and extended those observations in JEG3 cells by transiently transfecting them with CRH-luciferase expression vector. We treated the cells with different concentrations of cAMP (0.1, 0.5, 1 μM) for 5 or20 hours and observed that cAMP treatment increased CRH promoter activation in a dose dependent manner and this was evident at 5 h (data not shown). Based on this data we stimulated the cells with 0.5 μM cAMP for 5 hr in the rest of the experiments. The transcription factor AP-1 has been shown to mediate cAMP induced CRH promoter activation in humans [13]. As anticipated cAMP stimulation induced luciferase activity significantly above media treated control in JEG3 cells transiently transfected with AP-1 luciferase vector (Figure 1). Pair-wise comparisons using Tukey's test found that this increase was maximal at 0.3 μM cAMP concentration.

### DN-MyD88 and DN-TRIF block the cAMP induced CRH promoter activation in JEG3 cells

Infection induced innate immune responses play a significant role in the pathogenesis of preterm delivery. Innate immune receptors, Toll like receptors, are expressed in the female reproductive system and placenta, and mediate the immune responses to intrauterine infections [14,15]. MyD88 is the common adaptor molecule that transmits signals from all TLRs and the IL1 receptor to induce NF-κB activation [3,16].

We have previously shown that MyD88 mediates the LPS-induced, TLR4 mediated, CRH promoter activation in the placenta [7]. In order to assess whether MyD88 plays a direct role in cAMP-induced CRH promoter activation, we cotransfected JEG3 cells with CRH-luciferase and β-galactosidase expression vectors and either nonsignaling dominant negative (DN)-MyD88 [9] or empty vector and stimulated the cells with cAMP. There was no increase in cell death after transfection or cAMP treatment as assessed by microscope.

**Figure 2:**
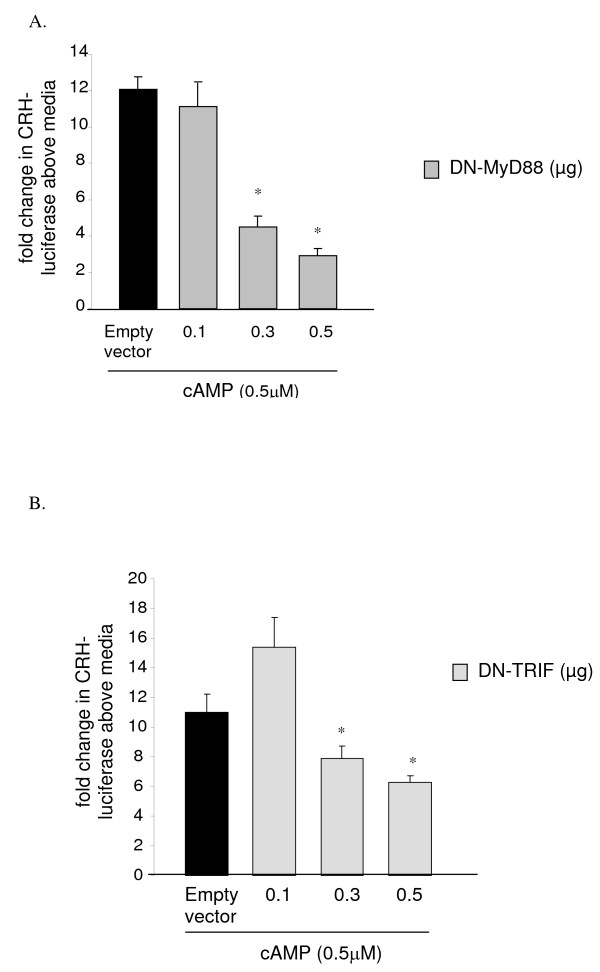
**We cotransfected the JEG3 cells with CRH-luciferase and B-galactosidase constructs and either empty vector (pcDNA3; 0.5 μg) or various concentrations of DN-MyD88 (Figure 2A) or DN-TRIF (Figure 2B) overnight using Fugene6**. The cells were treated with cAMP (0.3 μM) or media for 5 h. Luciferase activity was determined to assess CRH promoter activation. Calorimetric β-galactosidase assay was performed to correct for the transfection efficiency. (*p < 0.01 compared to empty vector transfected cells). The data was presented as fold change in luciferase activity above media treated-empty vector transfected control wells. Each experiment was performed in triplicate or quadruplicate and repeated at least three independent times.

We observed that expression of DN-MyD88 significantly blocked the cAMP-induced CRH expression in a dose dependent manner (Figure 2A). DN-MyD88 effect was maximal at 0.5 μM concentration, as the luciferase activity at this concentration was significantly lower than that seen with 0.3 μM DN-MyD88 (p = 0.03) (Figure 2A). These data suggest that inhibition of MyD88 signaling blocks the cAMP-induced CRH promoter activation.

MyD88-independent signaling is mediated via adaptor molecule TRIF, which transduces signals induced by TLR4 and TLR3 [3,17]. MyD88-independent pathways are involved in interferon (IFN) regulatory factor (IRF)-3 activation and subsequent induction of IFN-β and IFN-inducible genes and delayed NF-kB activation by TLR4. To test if TRIF plays a role in cAMP-induced CRH promoter activation, we cotransfected the JEG3 cells transiently expressing CRH-luciferase and β-galactosidase expression vectors with either DN-TRIF or empty vector and stimulated them with cAMP. Pair-wise comparisons using Tukey's test found that expression of both 0.3 and 0.5 μM DN-TRIF significantly inhibited the cAMP-induced CRH-luciferase expression (p < 0.01 for each concentration) (Figure 2B). This decrease was maximal at the 0.3 μM concentration of DN-TRIF (Figure 2B). These data suggest that inhibition of TRIF signaling blocks the cAMP induced CRH promoter activation.

IRAK2 is downstream to MyD88 and mediates the MyD88 induced NF-κB activation [3,9]. We transiently transfected the JEG3 cells expressing CRH-luciferase and β-galactosidase expression vectors with either empty vector or various concentrations of DN-IRAK2 cDNA. We stimulated the cells with cAMP for 5 hours and examined the CRH promoter activation by performing luciferase assays. We observed that expression of DN-IRAK2 cDNA did not block the cAMP-induced CRH promoter activation (data not shown). These data suggest that MyD88 regulation of cAMP-induced CRH expression is proximal to IRAK2.

### DN-MyD88 but not DN-TRIF blocks the cAMP induced CRE expression in JEG3 cells

**Figure 3:**
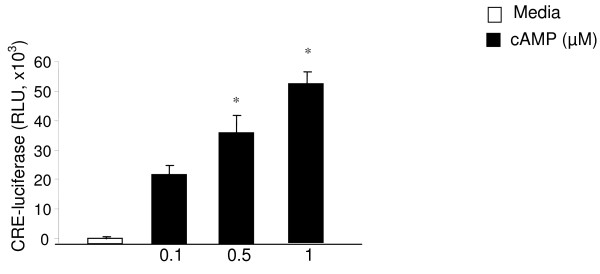
**The JEG3 cells were transiently transfected with CRE-luciferase and β-galactosidase reporter constructs overnight using Fugene6 (Roche) as described under Methods**. The cells were stimulated with either cAMP or media. Luciferase assay was performed to assess CRH promoter activation. Calorimetric β-galactosidase assay was performed to correct for the transfection efficiency. Luciferase activity was expressed as relative light unit (RLU). (*p < 0.01 compared to media treated cells). Each experiment was performed in triplicate or quadruplicate and repeated at least three independent times.

The cAMP response element (CRE) is downstream of PKA and mediates the cAMP induced CRH promoter activation in trophoblasts [1,8,18]. We questioned whether MyD88 and TRIF regulated cAMP-induced CRE promoter expression. We first confirmed that 5 hr stimulation with cAMP induces CRE activation in a dose dependent manner in JEG3 cells transiently expressing the CRE-luciferase expression vector (Figure 3). This increase was maximal at 0.5 μM cAMP as CRE-luciferase levels were significantly higher at this concentration over those seen at the 0.3 umol concentration (p < 0.01) (Figure 3).

**Figure 4:**
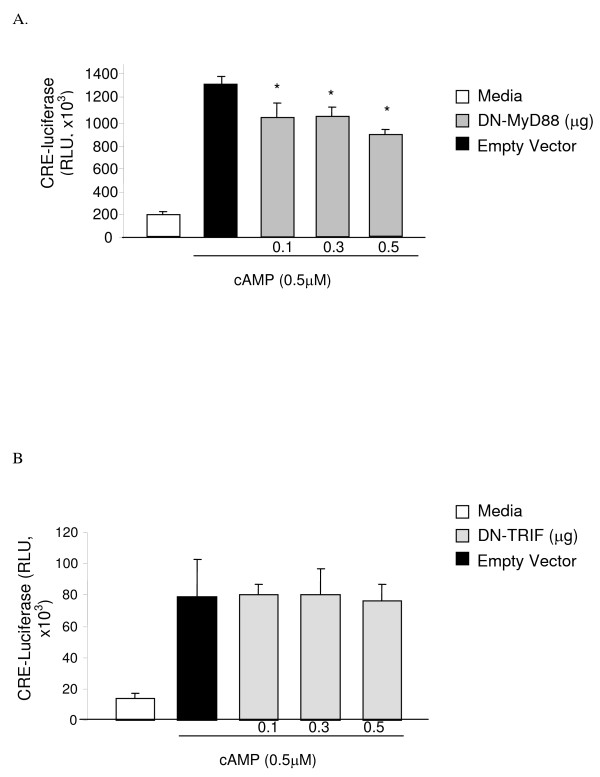
**We cotransfected JEG3 cells expressing CRE-luciferase and β-galactosidase expression vectors with empty vector (0.5 μg) and DN-MyD88 (0.1, 0.3, 0.5 μg) or DN-TRIF (0.1, 0.3, 0.5 μg) cDNA as described above, treated them with cAMP (0.5 μM) or media for 5 h, and assessed the luciferase activity**. Calorimetric β-galactosidase assay was performed to correct for the transfection efficiency. (*p < 0.01 compared the cAMP treated empty vector transfected control). Luciferase activity was expressed as relative light unit (RLU). Each experiment was performed in triplicate or quadruplicate and repeated at least three independent times.

Next, we tested whether Myd88 and TRIF influences cAMP-induced CRE expression. In JEG3 cells transiently cotransfected with CRE-luciferase vector, expression of DN-MyD88 cDNA significantly decreased the cAMP-induced CRE-luciferase levels in a dose dependent manner (p < 0.01 for all concentrations). This decrease was maximal at 0.5 μM concentration of DN-MyD88; at this concentration of DN-MyD88 cAMP-induced luciferase activity was significantly lower than that observed with the 0.3 μM concentration (p = 0.04) (Figure 4A). In contrast, DN-TRIF did not inhibit cAMP-induced CRE promoter activation (Figure 4B).

## Discussion

cAMP is a ubiquitous second messenger that regulates numerous biological processes. Here we show that the innate immune system molecules MyD88 and TRIF regulate the cAMP signaling in trophoblasts. In JEG3 cells, inhibition of MyD88 signaling blocked the cAMP-induced CRE-promoter and CRH-promoter expression; whereas inhibition of TRIF blocked only the CRH promoter activation.

cAMP is produced in response to hormones and nutrients, and via PKA regulates numerous processes (i.e. cardiovascular function [19], glucose homeostasis [20], adipocyte metabolism [21], growth-factor-dependent cell survival [22], learning and memory [23], immune function [24] and exocytotic processes such as gastric acid secretion [25]). cAMP also regulates reproductive function. Acute steroid biosynthesis is regulated by cAMP induced cholesterol release from lipid droplets and cholesterol transport across the mitochondrial membrane [26]. The initiation and maintenance of sperm motility depends on cAMP and PKA [27]. The gonadotropin induction of ovulation and oocyte maturation are associated with increased cAMP levels in the ovarian follicles [28].

During pregnancy, cAMP has diverse functions. Through its effects on calcium and potassium channels and myosin light chain kinase, cAMP promotes myometrial relaxation [29]. Tocolytic beta-mimetics operate through cAMP to inhibit uterine contractility in preterm labor [30] whereas in the trophoblasts, cAMP induces the release of CRH. CRH then crosses into the fetus to induce dihydroepiandrosterone (DHEA) release. DHEA is converted into estrogen in the placenta, and estrogen then induces the expression of genes that lead to cervical softening and myometrial contractility [31]. CRH expression has been shown to be higher in women who deliver preterm and increased CRH levels have been proposed to play role in early parturition [32].

In the placenta, cAMP induces CRH expression through activation of PKA, CRE and transcription factor AP-1 [1,8,18]. Here we first confirmed that cAMP induces CRH promoter activation through CRE and AP-1 in the JEG3 cell line and showed that innate immune system molecules, MyD88 and TRIF, play a direct role in cAMP-induced CRH promoter activation in JEG3 cells. Our data suggests that IRAK2 does not play a role in MyD88 regulation of cAMP signaling.

Adenylate cyclases (AC) synthesize cAMP from ATP and these enzymes are found in microbes as well as humans. In microbes, cAMP signaling is involved in the pathogenesis and virulence by regulating microbial metabolism, stress resistance, and maturation [reviewed in [33]]. Our data potentially suggest that the innate immune system molecule, MyD88, may regulate microbial-cAMP signaling and may potentially induce a direct antimicrobial effect. Cirl and colleagues have recently shown that virulent bacteria evolved a mechanism to inhibit the host MyD88 specific signaling to suppress host innate immunity [34]. We propose that microbial pathogens may potentially inhibit host cell MyD88 signaling to suppress cAMP signaling to regulate host metabolism, immunity, and cardiovascular function.

cAMP regulates gene transcription via PKA. In the basal state, PKA resides in the cytoplasm as an inactive heterotetramer of paired regulatory (R) and catalytic (C) subunits. cAMP liberates the C subunits, which passively diffuse into the nucleus and phosphorylate CREB [35]. CREB mediates the activation of cAMP-responsive genes by binding as a dimer to a conserved cAMP-response element (CRE) [36].

**Figure 5:**
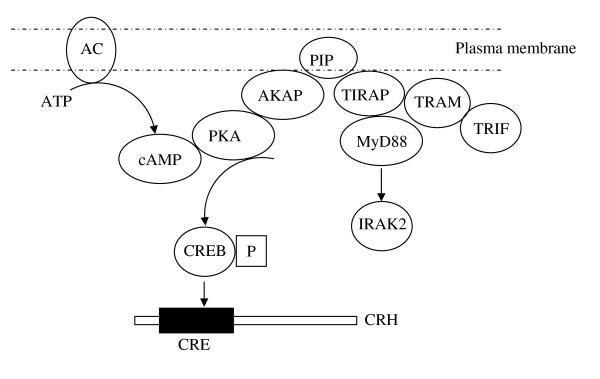
**Proposed Mechanism of MyD88 Regulation of cAMP signalling**. MyD88 interacts with TIRAP and PIP2 in the plasma membrane and associates with AKAP. AKAPs are known to regulate cAMP signaling through PKA. MyD88 may regulate cAMP signaling in a complex involving TIRAP-PIP2-AKAP. [ATP- Adenosine-5'-triphosphate; AC-adenylate cyclase; PIP2-phosphatidylinositol 4,5-bisphosphate; AKAP-A-kinase anchoring proteins; PKA-protein kinase A; CRE-cAMP-response element; TIRAP-TIR domain containing adaptor molecule; CREB-P-phosphorylated cAMP-response element binding (CREB)]

cAMP is known to inhibit immune activation in macrophages since 1970s [37]. Scaffold proteins called A-kinase anchoring proteins (AKAPs) are known to mediate cAMP inhibition of immune activation via protein kinase A [38,39]. AKAPs also form complexes with other signaling molecules for specificity of signaling. For example, AKAP79 binds to PKA, protein kinase C (PKC), and protein phosphatase 2B. AKAP79 basic regions also bind to membrane vesicles containing acidic phospholipids including phosphatidylinositol-4, 5-bisphosphate [PtdIns(4,5)P2] [40]. MyD88 is recruited to TLR4 by TIRAP, which interacts with phosphatidylinositol-4,5-bisphosphate (PtdIns(4,5)P_2_) rich regions of the plasma membrane through its amino-terminal phosphatidylinositol 4,5-bisphosphate (PIP2)-binding domain [41]. MyD88 may potentially interact with an AKAP to regulate PKA function and cAMP induced signaling (Figure 5). Indeed MyD88 has been shown to contain a PKA binding site (personal communication with Dr. Douglas Golenbock, University of Massachusetts Medical School).

## Conclusion

Here we demonstrate that inhibition of MyD88 and TRIF signaling block cAMP-induced CRH promoter activation in the JEG3 cells in the absence of infection. These data add to our previous findings on infection induced placental CRH expression [7] and suggest a direct role for innate immune system adaptor molecules MyD88 and TRIF to regulate cAMP signaling in the absence of infection. Inhibitors of MyD88 signaling are considered as potential anti-inflammatory treatments; our data suggest that it is important to understand the effect of MyD88 on cAMP signaling.

## Abbreviations

(TLR): Toll like receptor; (AP-1): activator protein 1; (TIR): Toll/Interleukin-1 receptor; (TRIF): TIR-domain-containing adapter-inducing interferon-β; (TIRAP): TIR-domain-containing adaptor molecule; (MyD88): myeloid differentiation primary response; (CRH): corticotrophin releasing hormone; (AC): Adenylate cyclases; (cAMP): cyclic adenosine monophosphate; (PtdIns(4,5)P_2_): phosphatidylinositol-4,5-bisphosphate; (PIP2): phosphatidylinositol 4,5-bisphosphate; (AKAPs): A-kinase anchoring proteins; (PKA): protein kinase A; (CRE): cAMP-response element.

## Competing interests

The authors declare that they have no competing interests.

## Authors' contributions

AU conducted the transfection, luciferase and galactosidase experiments; CFS provided funds, helped with data analyses and manuscript preparation; CTB conducted the statistical analyses; AFG helped with the study design, data analyses, and manuscript preparation, he developed and provided help with the CRE and AP-1 vectors; HA helped with data analyses and manuscript preparation; RN developed the CRH-luciferase vectors, he helped with the study design, data analyses, manuscript preparation; HK and NK conducted the transfection, luciferase and galactosidase experiments; OE initiated and designed the project, analyzed the data, wrote the manuscript. All authors have read and approved the final manuscript.
